# Analysis of Heart-Sound Characteristics during Motion Based on a Graphic Representation

**DOI:** 10.3390/s22010181

**Published:** 2021-12-28

**Authors:** Chen-Jun She, Xie-Feng Cheng, Kai Wang

**Affiliations:** College of Electronic and Optical Engineering, Nanjing University of Posts and Telecommunications, Nanjing 210023, China; 2016020222@njupt.edu.cn (C.-J.S.); ise_wangk@ujn.edu.cn (K.W.)

**Keywords:** sound-direction vector, motion–response curve, motion heart sound, multivariate feature analysis

## Abstract

In this paper, the graphic representation method is used to study the multiple characteristics of heart sounds from a resting state to a state of motion based on single- and four-channel heart-sound signals. Based on the concept of integration, we explore the representation method of heart sound and blood pressure during motion. To develop a single- and four-channel heart-sound collector, we propose new concepts such as a sound-direction vector of heart sound, a motion–response curve of heart sound, the difference value, and a state-change-trend diagram. Based on the acoustic principle, the reasons for the differences between multiple-channel heart-sound signals are analyzed. Through a comparative analysis of four-channel motion and resting-heart sounds, from a resting state to a state of motion, the maximum and minimum similarity distances in the corresponding state-change-trend graphs were found to be 0.0038 and 0.0006, respectively. In addition, we provide several characteristic parameters that are both sensitive (such as heart sound amplitude, blood pressure, systolic duration, and diastolic duration) and insensitive (such as sound-direction vector, state-change-trend diagram, and difference value) to motion, thus providing a new technique for the diverse analysis of heart sounds in motion.

## 1. Introduction

The heart-sound signal is one of the most important physiological signals in the human body. The detection and analyses of heart sounds are an important and economical means to understand the physiological information of the human body and have proven to be valuable for disease detection and biometric identification [1,2,3,4,5,6,7,8,9]. The most common method for the extraction of heart sound features is time–frequency analysis, represented by the wavelet transform method [4,10,11,12], and power spectrum analysis, represented by the FFT method [13,14,15,16,17]. Moreover, according to the premise of these analyses, heart-sound signals must be collected in a resting state.

If the resting state is a special case of motion, then the heart sound in the resting state can also be defined as a special form of heart sound in motion. However, there are obvious characteristic differences according to the analysis of the time–frequency characteristics and power spectrum characteristics of heart-sound signals generated by the same subject in a resting state and a state of motion [18,19]. Nevertheless, the heart-sound signals generated in a resting state and the heart-sound signals generated in a state of motion are produced by the same heart. Thus, there must be some features that are not sensitive to the state of motion and preserve the invariance of features. Therefore, it is useful to study the various characteristics of heart-sound signals during a state of motion.

Several interesting studies have been conducted on motion and heart sounds. In [20], Guo X. et al. studied the heart-sound recognition method including exercise heart-sound data. A probabilistic neural network was used to identify heart sounds after resting and exercise load as well as normal and abnormal heart sounds, and the recognition result reached a 94% recognition rate. In [21], Wu W. Z. et al. believed that the amplitude ratio of the first heart sound to the second heart sound reflected the relationship between cardiac contractile force and peripheral resistance and that exercise heart sound could be used to analyze cardiac reserve characteristics. It is suggested that the ratio of cardiac sound amplitude to cardiac reserve, cardiac fatigue, and hypoxia is of important value. In [22], the cardiopulmonary exercise test (CPET) was used to study the influence of exercise on cardiopulmonary reserve function, and the problems of CPET guiding cardiac rehabilitation in patients with coronary heart disease were studied. It was believed that the cardiopulmonary exercise test guiding cardiac rehabilitation could significantly improve cardiopulmonary reserve function in patients with coronary heart disease. The relationship between the cardiac sound amplitude ratio and diastolic systolic ratio and cardiac reserve function was studied by exercise experiments in [23]. It is concluded that the effect of exercise on cardiopulmonary reserve function can be discussed by comparative analysis of resting and motion heart sounds. To sum up, it is of positive significance to study the influence of human motion on cardiac characteristics. At present, the research on heart sound under a stage of motion mainly focuses on the analysis of cardiac reserve characteristics, and many beneficial conclusions have been obtained. In order to expand the characterization methods of heart sounds during motion and to explore new techniques for analyzing heart sounds during motion, it is necessary to conduct in-depth research on the characteristics of heart sounds under a motion state from multiple perspectives.

Admittedly, it remains challenging to determine how to transform the eigenvalues of in-motion and resting heart sounds into appropriate graphs for illustration. The present research aimed to represent the characteristics of motion and resting heart sounds via pictorial methods and to analyze the similarities and differences between in-motion and resting heart sounds from different angles. We first introduce a shoulder-strap-type single-channel wireless heart-sound collector and a shoulder-strap-type four-channel wired heart-sound collector and then examine the multiple characteristics of the single-channel heart-sound signal from a resting state to a state of motion. We further analyzed multi-channel heart-sound signals using the graphic representation method, because multi-channel heart-sound signals provide more overall information about the heart than that provided by a single-channel heart-sound signal [3,15,24]. Therefore, based on the idea of integration, we explored a multi-characteristic-representation method for studying the motion of heart sounds.

Several new concepts, such as the direction vectors of sounds, the motion–response curves of heart sounds, the difference value, and trend diagrams of status changes, are also proposed. Further, based on the acoustic principle, the reasons underlying the differences between the multi-channel heart-sound signals were analyzed. Finally, several feature parameters that are sensitive and insensitive to motion are provided through verification using a simulation experiment and graphical representation methods. The above research provides a new technique for analyzing the diverse characteristics of heart sounds under a state of motion.

## 2. Experimental Platform and Method

### 2.1. Heart-Sound Collector

**Figure 1:**
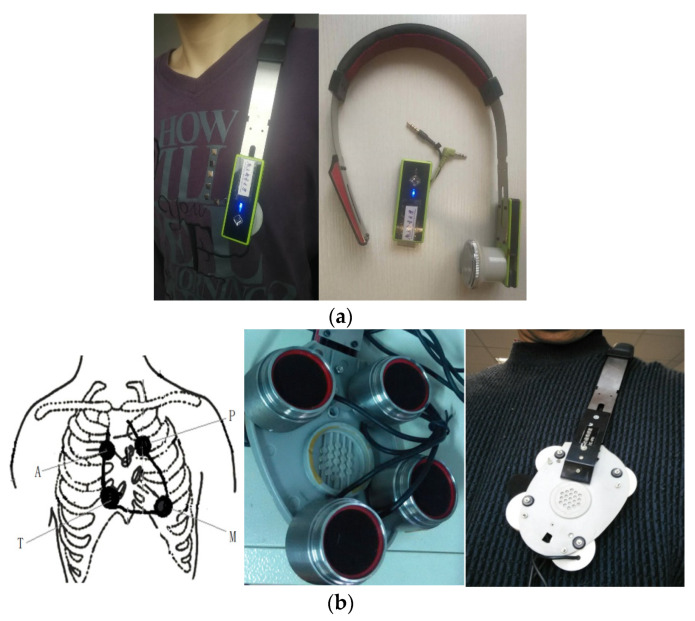
Single-channel and four-channel heart-sound collectors: (**a**) shoulder-strap-type single-channel wireless heart-sound collector; (**b**) schematic diagram of 4 auscultation areas and 4-channel heart-sound collectors.

Since a heart sound is an extremely weak acoustic signal, our single-channel heart-sound sensor was used for blood-pressure monitoring [25] and identification [1]. At the same time, we designed a four-channel heart-sound collector based on the design of a mature single-channel heart-sound sensor. A rapid-screening instrument for congenital heart disease in children based on four-channel heart-sound acquisition is clinically used for screening neonatal congenital heart disease at Shanghai Xinhua Hospital. To collect heart sounds in the present research, we used a shoulder-strap-type single-channel wireless heart-sound collector (patent No.: ZL201310454575.6) and a shoulder-strap-type four-channel wired heart-sound collector (patent No.: ZL201310454575.6), which were both designed and produced by the research group (as shown in Figure 1a,b). The images in Figure 1 were created by us; the devices were also made by the team and are patented. The content of the logo in Figure 1a is the name of our college, which we have the right to use: Nanjing University of Posts and Telecommunications (in Chinese). The device consists of lightweight elastic materials that form an Ω frame component which has a shape that is similar to that of a human shoulder on the side of the chest outside the contour lines, thus allowing the device to be easily placed on the left shoulder. Moreover, the length of the end of the Ω frame can be partially adjusted to ensure that the heart-sound sensor can accurately remain in the auscultation position of a human heart. The shoulder-strap-type single-channel wireless collector has a heart-sound sensor installed only on the Ω frame at the end, and the 4-channel shoulder-strap-type collector has four heart-sound collectors installed in the Ω frame at the end. The end is a heart-shaped base made of lightweight elastic materials, such as metal and plastic, and the base is actually larger than an actual heart. The four heart-sound sensors are arranged in accordance with the positions of the four auscultation areas as shown in Figure 1b. These areas include the aortic auscultation area (A), pulmonary auscultation area (P), tricuspid auscultation area (T), and mitral auscultation area (M) corresponding to the chest areas on a human body. The sensors seek to obtain the heart-sound signals at the corresponding positions called the A-channel, T-channel, P-channel, and M-channel signals. Then, the heart sounds are collected in real time via wireless and wired means, thus providing a variety of acquisition methods for heart sounds in a state of motion.

### 2.2. Data Collection Methods

The step-test method was adopted in this paper. The heart-sound collection experiment in this project was approved by the Biological and Medical Ethics Committee of Dalian University of Technology. The subjects were informed of the content, purpose, and matters requiring attention before the experiment and signed their informed consent. The step height for males was 30 cm, and the step height for females was 25 cm; the rhythm was 30 times per minute (up and down) for a total of 3 min. The procedures of heart-sound collection based on the step-test method were as follows:

Step 1: Resting state—researchers collected the heart-sound signals of the tester for 10 s; this kind of signal is represented by Step 1:A;

Step 2: State of motion—The test subjects took the step test. Immediately after the first minute of the test, the subjects stopped moving; 10 s of heart-sound signals and blood pressure readings were collected synchronously. This kind of signal is represented by Step 2:A. Immediately after the second minute of the test, the subjects stopped moving, and the heart-sound signals and blood pressure were collected for 10 s synchronously; these signals are represented by Step 2:B;

Step 3: Post-exercise state—At the second minute after the step test, the heart-sound signal and blood pressure were collected for 10 s synchronously. Such signals are represented by Step 3:A. In addition, the same procedures were carried out on the 12th and the 22nd minutes and were designated as Step 3:B and Step 3:C, respectively.

Heart-sound data were collected in a natural environment by our research group. Thirty-five subjects (25 males and 10 females, aged 20–65, 155–185 cm in height, and 45–90 kg in weight) were selected to participate in the experiment. The 35 subjects were divided into 7 groups with 5 members in each group and numbered (e.g., the second subjects in the first group were defined as 1–2). All subjects were healthy and did not have any major illnesses. The subjects were asked to avoid caffeine, alcohol, and cigarettes for two hours before the study.

Ultimately, we collected a total of 162 groups of heart sounds in a state of rest, 148 in a state of motion, and 148 in a state of recovery after exercise.

**Figure 2:**
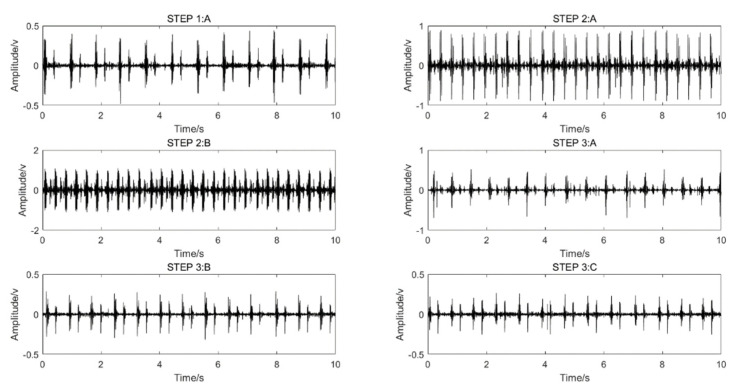
Single-channel heart-sound signals in different states of motion: resting state—Step 1A; state of motion—Step 2A,B; post-exercise state—Step 3A–C.

**Figure 3:**
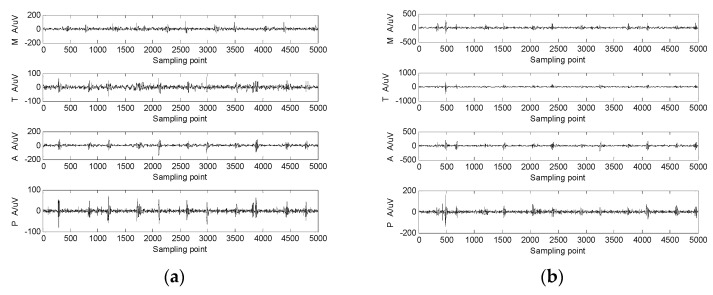
The 4-channel heart-sound signal in 2 states: (**a**) resting state—Step 1A; (**b**) state of motion—Step 2A.

A shoulder-strap-type single-channel wireless heart-sound collector was used to collect the heart-sound signals of the test subjects according to the above experimental procedures. The waveforms of the heart-sound signals within each group are shown in Figure 2. The four-channel heart-sound collector was used to collect the heart-sound signals of the test subjects in accordance with the above experimental procedures. The waveforms of heart-sound signals for one set (Step 1:A and Step 2:B) are shown in Figure 3.

## 3. Analysis Results for the Characteristics of Heart Sounds in Motion

### 3.1. Basic Model and Characteristics of Heart-Sound Signals

A heart-sound signal includes five main components [1,4]. (1) The first heart-sound (S1) signal, occurring in the ventricular systolic period, has a low pitch, a relatively loud sound, and a relatively long time span lasting approximately 0.1~0.12 s, with a frequency of 40~60 Hz. (2) The second heart-sound (S2) signal occurs in the ventricular diastole, with a high pitch and short duration lasting approximately 0.08 s and a frequency of 60~100 Hz. (3) The third heart-sound (S3) signal, which has a weak intensity, is produced in the rapid filling period during the early ventricular diastolic stage and is often seen in the heart sounds of children and adolescents. (4) The fourth heart-sound (S4) signal occurs in the late ventricular diastole stage and appears in people over the age of 40. (5) A heart-murmur signal ($w_{s}$) refers to a type of long-lasting non-heart sound (other than the heart sound and additional heart sound) with different frequencies and intensities. Usually, $s_{3}$ and $s_{4}$ are weak and difficult to collect, and only $s_{1}$, $s_{2}$, and $w_{s}$ can be collected under normal circumstances. According to the single-channel heart-sound signal model [1], we determined that N-channel heart-sound signals in one cycle, T, can be described using Equation (1):

(1) $$S_{T}^{n}\left(t\right)=\left[\begin{array}{c} k_{11}s_{1}^{11}\left(t\right)+k_{12}s_{2}^{12}\left(t\right)+k_{13}s_{3}^{13}\left(t\right)+k_{14}s_{4}^{14}\left(t\right)+k_{15}w_{s}^{15}\left(t\right)\\ k_{21}s_{1}^{21}\left(t\right)+k_{22}s_{2}^{22}\left(t\right)+k_{23}s_{3}^{23}\left(t\right)+k_{24}s_{4}^{24}\left(t\right)+k_{25}w_{s}^{25}\left(t\right)\\ \vdots \\ k_{n1}s_{1}^{n1}\left(t\right)+k_{n2}s_{2}^{n2}\left(t\right)+k_{n3}s_{3}^{n3}\left(t\right)+k_{n4}s_{4}^{n4}\left(t\right)+k_{n5}w_{s}^{n5}\left(t\right) \end{array}\right]$$

where $s_{1}$ and $s_{2}$ represent the first and second heart-sound signals, respectively; $s_{3}$ and $s_{4}$ represent the third and fourth heart-sound signals, respectively, which are generally not discussed. $w_{s}$ represents a murmur in the heart sound, and k represents the synthesis factor. This is the basic model of a multi-channel heart-sound signal.

A heart-sound signal is related to each individual’s heart location, size, chest structure, age, gender, weight, mood, motion status, and other factors, leading to differences in the heart-sound signals of each person. However, under relatively fixed conditions, a single person’s heart-sound signals will remain relatively stable and unchanged over a long time range. The heart-sound signal has the following basic characteristics.

Heart-sound signals are represented in a narrow frequency band. The frequency band of heart-sound signals usually ranges from 0 to 600 Hz. The frequency component of the first heart sound is mainly concentrated in the range of 50 to 150 Hz, while the frequency component of the second heart sound is mainly concentrated in the range of 50 to 200 Hz. A second small peak, the amplitude of which changes significantly, appears in the range of 250 to 350 Hz, while the other frequency ranges are close to zero. The amplitudes of these bands change significantly, while the other frequency ranges trend toward zero. Exercise shifts the frequency component of the heart-sound signal to a higher location.

Heart-sound signals feature quasi-periodic repeatability. However, the differences between the periodic waveforms are very small and are usually processed as periodic stationary repetitive signals. Exercise, moreover, shortens the period of a heart-sound signal.

A heart-sound signal is also characterized by its energy concentration. The energy of heart-sound signals is mainly distributed in the first heart sound and second heart sound, which represent the main positions to extract the characteristic parameters of heart sounds. Exercise also increases the energy of a heart-sound signal.

### 3.2. Influence of Motion on the Sound-Direction Vector of Heart Sounds

As a kind of periodic mechanical vibration, the heart sound, which has its own mechanism, provides a reflection of the characteristics of the myocardium and heart valve as well as the walls of large vessels. There are corresponding division standards and specific forms of expression for the representation of heart sounds in motion.

**Definition** **1.** *P–P–F phonocardiogram. The variation rule of the power and frequency of heart-sound signals within a given period are marked in the improved polar coordinate diagram and defined using a period–power–frequency (P–P–F) phonocardiogram.*

The method for obtaining a P–P–F phonocardiogram is illustrated as follows. For any heart-sound signal $s^{n}\left(m\right)$ (n=1,⋯,N;m=1,⋯,M), the number of channels for the heart sounds collected is marked as N, where M is the length of the signal, and $s^{n}\left(m\right)$ is the heart-sound signal of channel n. Fourier transform can then be used to obtain the heart-sound’s power spectrum characteristics using Equation (2):

(2) $$\begin{array}{c} P=e^{\frac{\left|W\left(m\right)\right|}{{\left|||W\left(m\right)||\right|}_{\infty }}}\\ F\left(m\right)=2\pi mf_{h}/M\;\;\;m=0,\cdots ,M \end{array}$$

where $W\left(m\right)=\overset{M}{\underset{m=0}{\sum }}{\left|s^{n}\left(m\right)\right|}^{2}e^{j2\pi f_{h}}$, and $f_{h}$ is the upper limit frequency of heart-sound signals. Although the spectrum of heart sounds is concentrated at 10–200 Hz, full recognition requires more information, so $f_{h}$ ≥ 540 Hz is commonly taken. The assumptions are as follows:

(3) $$\begin{array}{c} x=P\cos(F)\\ y=P\sin(F) \end{array}$$

Within a range of 0∼2π, we used the improved polar coordinate method to draw the frequency coordinate of a period and then drew *x* and *y* on that coordinate. Subsequently, an arrow was used to represent the frequency coordinate by taking $f_{h}$/2π as Hz/degrees, thus obtaining a P–P–F phonocardiogram. The P–P–F phonocardiogram has the following characteristics: (1) It reflects the relationship between the normalized power and frequency of a periodic heart-sound signal and iteratively describes the change process of that signal on the same circle. Visual comparison clearly highlights the detailed differences in the process of change and effectively realizes a periodic description of the heart-sound signal. (2) During the process of analyzing the power spectrum of a heart-sound signal, the signal is normalized and then exponentially processed. The purpose of this process is to magnify the details approaching zero and compress the maximum value within 1. (3) The heart-sound signal is a nonlinear time-varying signal. Thus, the same person’s heart-sound signal will be different before and after exercise. The P–P–F phonocardiogram can effectively reflect this phenomenon. (4) Frequency murmurs and amplitude distortions in heart sounds can be characterized by a comparative analysis of the P–P–F diagram.

**Definition** **2.** *Similarity distance (SD). The heart sound in a resting state is set as $C_{i}\left(t\right)$, and the heart sound in a state of motion is set as $s_{j}\left(t\right)$. Then, the similarity distance is expressed by the following Equation (4):*

(4) $$SD=1-\frac{\left|\sum_{t=1}^{N}c_{i}\left(t\right)s_{j}\left(t\right)\right|}{\sqrt{\sum_{t=1}^{N}c_{i}^{2}\left(t\right)\sum_{t=1}^{N}s_{j}^{2}\left(t\right)}}$$

where *i* and *j* are the labels of the number of segments of the heart-sound signal. Next, the two signals are aligned, and then Equation (4) is used to calculate the similar distance. Here, the smaller the similarity distance SD is between $C_{i}\left(t\right)$ and $s_{j}\left(t\right)$, the greater the similarity is between $C_{i}\left(t\right)$ and $s_{j}\left(t\right)$. When SD is equal to 0, $C_{i}\left(t\right)$ = $s_{j}\left(t\right)$, which means 100% similarity.

**Definition** **3.** *Similarity phase diagram. The lattice diagram of the two signals in the phase space is defined using a similarity phase diagram.*

This method can visually show the amplitude difference and phase difference between $C_{i}\left(t\right)$ and $s_{j}\left(t\right)$ and is able to comprehensively analyze and evaluate $C_{i}\left(t\right)$ and $s_{j}\left(t\right)$ with the assistance of the similarity distance.

The judgment rules for similarity phase diagrams are as follows:

The amplitude of $C_{i}\left(t\right)$ is set as A1, the frequency is set as ω1, and the phase angle is set as φ1. Moreover, the amplitude of $s_{j}\left(t\right)$ is expressed as A2, the frequency is set as ω2, and the phase angle is defined as φ2.

Rule 1. If A1 = A2, φ1 = φ2, and ω1 = ω2, then the similarity phase diagram is an oblique line with an angle of 45° located in the first phase limit, where SD = 0;

Rule 2. If A1 = kA2, φ1 = φ2, and ω2 = ω1, then the similarity phase diagram is a thick oblique line of width d with an angle of 45°, and d∝k, where SD ≈ 0;

Rule 3. If A1 = A2, ω2 = ω1, and φ1 − φ2 = Δφ, fine diagonals no longer create a 45° angle but change along with a change of Δφ. When Δφ = 180°, SD = 0. The similarity phase diagram here is an oblique line with an angle of 135° in the second phase limit;

Rule 4. If A1 ≠ A2, ω2 ≠ ω1, and φ1 ≠ φ2, multiple closed chaotic graphs will appear. When the chaotic graphs fill the whole picture, SD ≈ 1.

**Definition** **4.** *The sound-direction vector of a heart sound. To highlight the invariance of heart sounds both at rest and in a state of motion, the sound-direction vector of heart sounds was introduced based on the acoustic concept. Since heart-sound signals are sparse, each heart-sound signal is self-convolved. Then, the signal’s frequency characteristics are analyzed, and an FFT map is produced; this map is called a sound-direction vector map of the heart sound. The horizontal axis represents the frequency, and the vertical axis represents the amplitude. When a resting heart sound becomes an in-motion heart sound, the peak point in the figure remains static. Here, the eigenvalue remains basically unchanged, highlighting the invariance of in-motion and at-rest heart-sound signals. The process for finding the direction vector of heart sounds is similar to that for finding the cepstrum. The cepstrum can help characterize the frequency periodicity of the signal, while the direction vector of heart sounds can help characterize the invariance of in-motion and at-rest heart-sound signals.*

The method for obtaining the sound-direction vector of heart sounds is as follows:

(1) Let the heart sound in a state of motion be $s_{j}\left(t\right)$, which is a sparse vector. Then, we obtain Equation (5):

(5) $$H_{j}=s_{j}\left(t\right)*s_{j}\left(t\right)$$

(2) Calculate the frequency response characteristics of $H_{j}$ and obtain the frequency response vector *A* (ω) and the corresponding angular frequency vector φ(ω).

(3) Then, perform a centrally symmetrical FFT analysis for *A* (ω) using Equation (6):

(6) $$Y_{j}=\frac{F\left[\left|A\left(\omega \right)\right|\right]}{e}$$

where $Y_{j}$ represents the sound-direction vector of heart sounds, e is a constant, and the graph drawn represents a sound-direction vector diagram of the heart sound.

**Figure 4:**
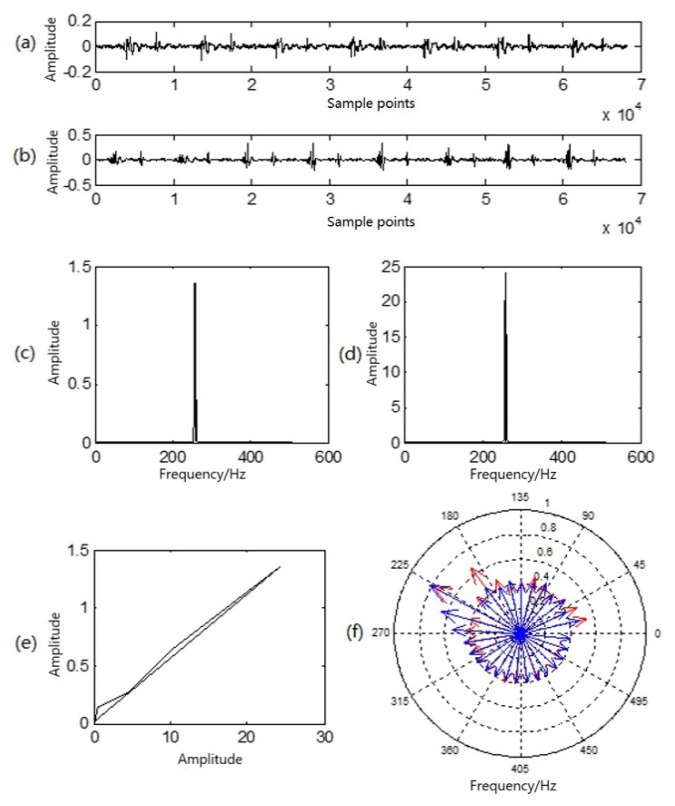
Multi-characteristic analysis diagram of a group of heart sounds in a resting state and a state of motion: (**a**) resting heart-sound signals; (**b**) in-motion heart-sound signals; (**c**) sound-direction vector of resting heart sounds; (**d**) sound-direction vector of heart sounds in motion; (**e**) similarity phase diagram of at-rest and in-motion heart sounds; (**f**) P–P–F heart sounds for at-rest heart sounds (red) and heart sounds in motion (blue).

Figure 4 illustrates comparative analyses of various characteristics of heart sounds at rest and in a state of motion.

Figure 4a illustrates a waveform of heart sound $C_{i}\left(t\right)$ in a resting state; Figure 4b is a waveform of heart sound $s_{j}\left(t\right)$ in a state of motion; Figure 4c is the sound-direction vector $Y_{i}$ of the heart sound in a resting state; and Figure 4d is the sound-direction vector $Y_{j}$ of the heart sound in state of motion. In Figure 4e, the similarity phase diagram of the two is an oblique line in the first phase limit with an angle of approximately 45°, and the similarity distance was 0.0038. It can be seen that the sound-direction vector of the two was basically unchanged, reflecting that the heart is the sound source and that motion exerts no influence upon the physical acoustic characteristics of the sound source itself. In Figure 4f, the red lines with arrows in the P–P–F phonocardiogram represent the heart sound in a resting state, while the blue lines represent the heart sound in a state of motion. Obviously, there are differences in the heart-sound signals before and after exercise, but the amplitude and frequency of the two at the highest point of the amplitude almost overlap, which corresponds to the peak in the sound-direction vector. As a result, the P–P–F phonocardiogram effectively and intuitively reflects this difference. Moreover, motion here shifted the overall P–P–F phonocardiogram to a higher location.

### 3.3. Influence of Motion on the Time-Domain Characteristics of Heart Sounds and Blood Pressure

Using the method proposed by Springer [4], we divided the duration of a cycle of heart-sound signals into four parts: S1 duration, systolic duration, S2 duration, and diastolic duration. The systolic, diastolic, first-heart-sound, and second-heart-sound duration in the literature is shown in [4]. The basic time-domain characteristics of heart-sound signals mainly include S1 amplitude, S2 amplitude, systolic duration, diastolic duration, and heart rate. To analyze the effects of motion on the heart sound and cardiovascular system from multiple angles, a routine characteristic parameter, blood pressure, was introduced. The calculation method for blood pressure used in this paper was taken from the method in [21].

#### 3.3.1. Influence of Motion on Heart-Sound Amplitude and the Corresponding Change Rule of Blood Pressure

Test subject number 1_5 was used as an example to analyze the heart-sound signals obtained by applying Steps 1–3 of the experiment.

**Figure 5:**
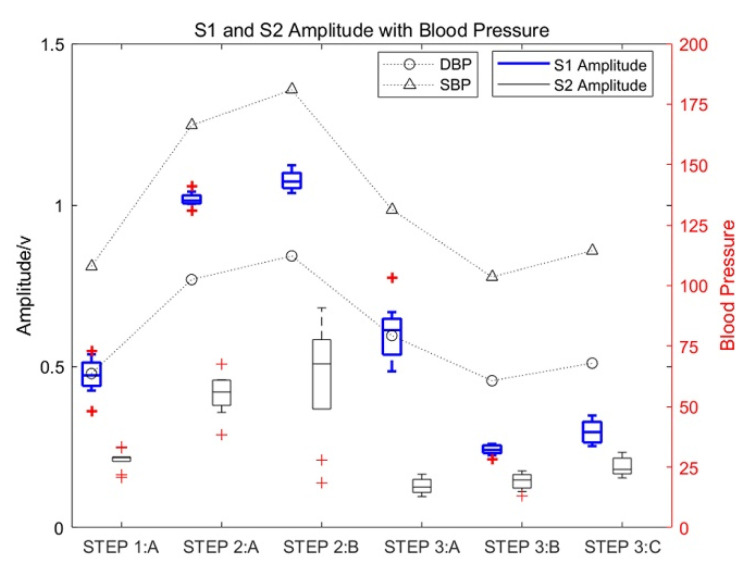
The influence of motion on the amplitude of heart sounds and the corresponding change rules of blood pressure.

Test subject 1_5 repeated the experiment 10 times for different time periods. Based on the results of the 10 experiments, the heart-sound signals of 10 cardiac cycles under different states of motion were selected for the box diagram analysis to analyze the influence of motion on the amplitude of heart sounds and the change rules of the corresponding blood pressure. The obtained experimental results are shown in Figure 5.

As shown in Figure 5, the amplitude of S1 and S2 increased significantly with an increase in the exercise intensity of test subject 1_5. The increase in S1 was especially significant, with the maximum increase being approximately three times the amplitude of S1 in a resting state, which gradually decreased with an increase in rest time. Moreover, the amplitude of S2 in a state of motion increased significantly, with the maximum increase approximately twice that of heart-sound S2 in a resting state. At the same time, the systolic pressure (SBP) and diastolic pressure (DBP) also increased with an increase in exercise intensity.

#### 3.3.2. Influence of Motion on the Diastolic and Systolic Duration of Heart Sounds and the Corresponding Change Rules of Blood Pressure

**Definition** **5.** *Motion–response curves of heart sounds. In a heart-sound signal, the difference between the diastolic and systolic ratios of any two different heart-sound cycles is called the ratio difference of diastolic/systolic heart sounds.*

In accordance with the collection procedures for heart sounds, the diastolic/systolic ratio difference of the heart-sound cycle between Step 1 and Step 2 is expressed using Equation (7):

(7) $${\mathrm{DS}D}_{k}=\begin{array}{cc} \frac{D_{i}}{S_{i}}-\frac{D_{i+1}}{S_{i+1}} & k=1,2\cdots 5 \end{array}.$$

**Figure 6:**
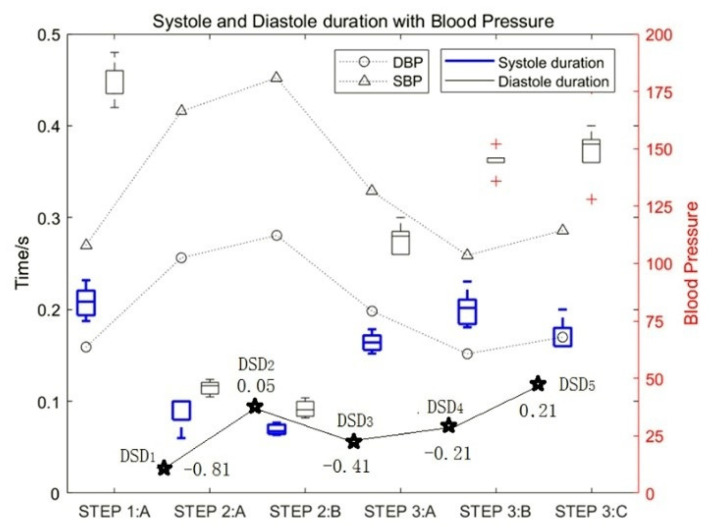
The influence of motion on the duration of diastolic and systolic heart sounds and the DSD values.

The curve composed of $DSD_{k}$ is defined as the motion–response curve of heart sounds, where *D* represents the duration of the diastole, *S* represents the duration of the systole, i marks the label of the collection step, and *k* represents the serial number for the value of *DSD*, *k* = 1, 2…5. Usually, the motion–response curves of an athlete’s heart sounds are relatively smooth, while the motion–response curves of those who do not often engage in exercise fluctuates greatly. The motion–response curves of heart sounds for test subject 1_5 are shown as the curves of point ★ in Figure 6.

The experimental data of test subject 1_5 was also used in this study. Figure 6 shows the influence of motion on the duration of diastolic and systolic heart sounds and the corresponding change rules of blood pressure. It can be seen that with an increase in exercise intensity, the duration of both the systolic and diastolic period decreased gradually, and the proportion of systolic periods and diastolic periods decreased by approximately 76% and 81%, respectively. In contrast, with an increase in rest time, the duration of both systolic and diastolic periods increased gradually. At the same time, the SBP and DBP increased with an increase in exercise intensity. 

**Figure 7:**
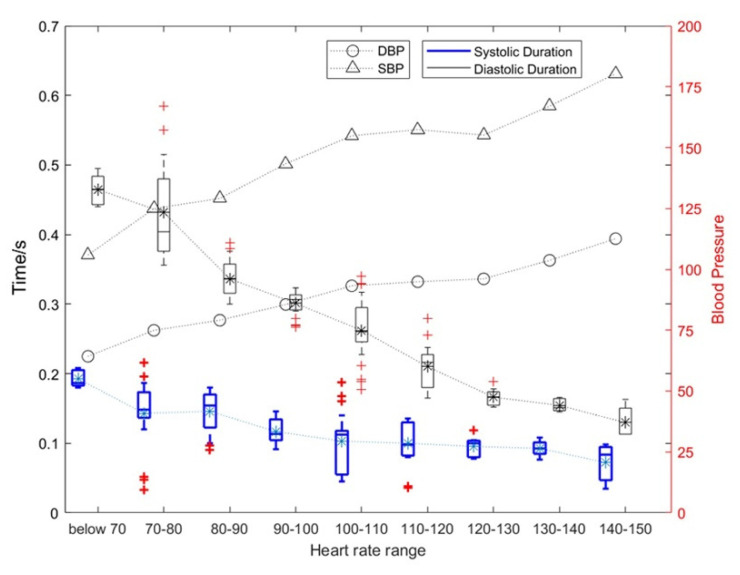
The influence of heart rate changes on the duration of diastolic and systolic periods and the corresponding blood pressure change rules.

The test data for all subjects were divided according to the subjects’ different heart-rate intervals. The influence of heart-rate changes on the duration of the diastole and systole and the corresponding change rules of blood pressure are shown in Figure 7. Figure 7 shows the results of the overall analysis.

As shown in Figure 7, as the heart rate increased, and the duration of systolic and diastolic periods gradually decreased. The average heart rate of a normal person is 75. When the heart rate exceeds 100, the heart can be considered to enter a state of motion; when the heart rate exceeds 120, the heart can be considered to enter a stronger state of motion. Under this stronger state of motion, the relative rate of change for the systolic period and diastolic period can reach 31.89% and 15.34%, respectively. The specific results are shown in Table 1. At the same time, the SBP and DBP also increase with an increase in exercise intensity with the highest being approximately 1.5 times the blood pressure at rest.

In addition, the heart rate in Figure 7 was *p*-value assessed against SBP, DBP, and diastolic and systolic durations. Among them, heart rate and SBP: *p* = 4.8316 × 10^−7^ < 0.01; heart rate and DBP: *p* = 2.0378 × 10^−6^ < 0.01; heart rate and systolic duration: *p* = 6.6777 × 10^−6^ < 0.01; heart rate and diastolic duration: *p* = 3.2495 × 10^−6^ < 0.01. This shows that these four features of the heart change as the heart rate changes, and they are significantly correlated.

As can be seen from Table 1, (1) the mean systolic time of the silent heart-sound signal was between 0.1171 and 0.1421 s, and the mean diastolic time was between 0.2991 and 0.3909 s; (2) the mean systolic time and the mean diastolic time were 0.0571–0.1012 and 0.2008–0.2544 s, respectively; (3) the number of motor heart-sound signals with the heart rate range over 120+ was relatively small, so they were combined into the heart rate range of 120+.

### 3.4. Influence of Motion on the Nonlinear Characteristics of Heart Sounds

#### 3.4.1. State-Change Trend Diagram of Heart-Sound Signals

**Definition** **6.** *State-change-trend diagram of heart-sound signals. The correlation degree and change trends for the multi-channel heart-sound signals in the phase space at rest and in a state of motion are reflected in the state-change-trend chart of heart-sound signals. This chart illustrates the morphological differences of multi-channel heart-sound signals in phase space with changes in the hysteresis coefficient.*

The method for obtaining the state-change-trend diagram of heart-sound signals is as follows:

(1) The multi-channel heart-sound signal $S_{T}^{n}\left(t\right)$ is normalized to obtain $P_{k}$ (*k* = 1,2,3,4);

(2) By setting the hysteresis coefficient as *m* (*m* = 1, 2..., *M*), *M* = 5, and *L* as the length of $P_{k}$, we obtain Equation (8):

(8) $$\{\begin{array}{c} {PP}_{1}\left(m\right)=\overset{L}{\underset{i=1}{\sum }}(P_{k}\left(i\right)-m)*\left(P_{k}\left(i+M\right)-m\right)\\ {PP}_{2}\left(m\right)=\overset{L}{\underset{i=1}{\sum }}(P_{k}\left(i\right)-m)*\left(P_{k}\left(i\right)-m\right)\; \end{array}\begin{array}{cc} , & \begin{array}{c} m=1,2\cdots 5\\ k=1,2\cdots 4 \end{array} \end{array}$$

(3) By taking the average value of *PP*_1_ and *PP*_2_, we obtain the difference value of the state change displayed in Equation (9):

(9) $$\mathrm{DS}\left(m\right)=\begin{array}{cc} \frac{\sqrt{\left|{PP}_{1}\left(m\right){-PP}_{2}\left(m\right)\right|}}{\sqrt{\left|{PP}_{1}\left(m\right){+PP}_{2}\left(m\right)\right|}} & m=1,2\cdots 5 \end{array}.$$

**Definition** **7.** *Difference value for the state changes of heart sounds. DS(m) is the specific parameter value in the trend diagram of the state change and is thus called DS(m) to reflect the difference value for the state change of heart sounds.*

(4) The two-dimensional diagram of DS(m) reflects the state-change-trend diagram of heart-sound signals.

**Figure 8:**
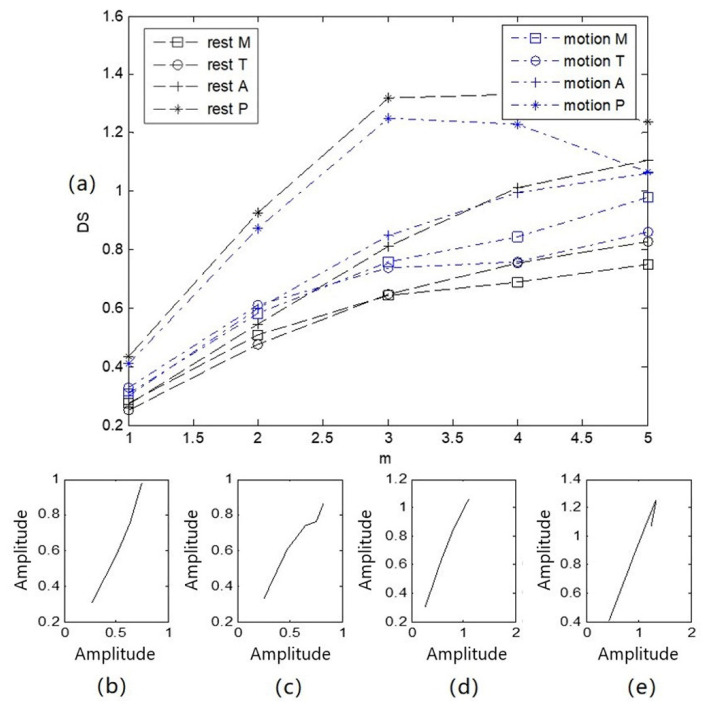
Trend diagram and similarity phase diagram of heart sounds in 2 states: (**a**) a state variation trend diagram of 4-channel heart-sound signals under 2 states; (**b**) similarity phase diagrams of Rest M and Motion M; (**c**) similarity phase diagrams of Rest T and Motion T; (**d**) similarity phase diagrams of Rest A and Motion A; (**e**) similarity phase diagrams of Rest P and Motion P.

The four channels of heart-sound signals (Step 1: A in a resting state and Step 2: A in a state of motion) in two states, as shown in Figure 3, were processed by obtaining the state-change-trend diagram of heart-sound signals. The obtained state-change-trend diagram is shown in Figure 8a. Figure 8b illustrates a similarity phase diagram of Rest M and Motion M; Figure 8c provides a similarity phase diagram of Rest T and Motion T; Figure 8d provides a similarity phase diagram of Rest A and Motion A; and Figure 8e provides a similarity phase diagram of Rest P and Motion P. Here, the M channel, T channel, A channel, and P channel represent four auscultation areas of the human body: the mitral valve auscultation area (M), pulmonary valve auscultation area (P), aortic valve auscultation area (A), and tricuspid valve auscultation area (T). Moreover, Rest represents a quiet state, and Motion represents a state of motion.

As shown in Figure 8, (1) due to the different auscultation positions of the four channels of the heart-sound signals, the state-change-trend chart for each channel is obviously different. This difference occurs because the thickness of the chest wall ranges from 1.1 to 3.7 cm, while the volume of the heart is approximately the size of a fist, and the average distance from the stethoscope to the heart is only about 3 cm. The heart as a whole cannot be equivalent to a point acoustic source; the local heart region corresponding to the auscultation area can only be equivalent to a point acoustic source. Thus, the four auscultation areas are equivalent to four different point-acoustic sources in the corresponding heart, and the state change difference value presented for each heart-sound signal is significantly different. (2) The heart-sound signals in a state of motion and a resting state were compared and analyzed. Rest P, motion P, Rest M, and motion M basically showed a parallel arrangement, with similarity distances of P = 0.0011 and M = 0.0006, respectively. Rest T, motion T, Rest A, and motion A first showed parallel arrangements and then intersected. At m = 4, the difference value of the state change was the smallest, and Rest T and motion T overlapped at this point, with similarity distances of T = 0.0038 and A = 0.0013, respectively. The maximum and minimum similarity distances in the corresponding state-change-trend graphs were found to be 0.0038 and 0.0006, respectively. This result indicates that the difference between similar distances was 0.0032. (3) The morphological difference value, as a characterization parameter for the nonlinear characteristics of heart sounds, is intuitive and comparable and can be used as a measure for the state changes of heart sounds.

#### 3.4.2. Attractor Phase Diagrams of Three States of Heart-Sound Signals

The modeling methods for heart sounds are discussed in [1], including the details Formula (1) and Formula (2) in the same paper. The heart-sound model in the present study was developed based on the methods outlined in the reference literature.

The nonlinear properties of heart sounds are demonstrated in [26,27,28]. Reference [26] demonstrated that the frequency characteristics of heart sounds are nonlinear. In [27], normal and pathological heart sounds were analyzed using the nonlinear characteristics of heart-sound signals. Reference [28] established a simulation model based on lumped parameters for the cardiovascular system; the authors also elaborated upon the mechanisms for heart-sound generation and cardiovascular coupling modes and demonstrated the relationship between the basic characteristics of heart sounds and cardiac physiological activity.

The cardiovascular system can be regarded as a nonlinear dynamic system and a heart-sound signal as a non-stationary signal [26,27,28]. The dynamic graph of a periodic heart-sound signal normally forms a regular and ordered ring. The motion track is recorded in the phase plane, which can reflect the change in heart-sound signals from a resting state to a state of motion. The dynamic change process of cardiac acoustic activities can be characterized from the perspective of time and space.

**Figure 9:**
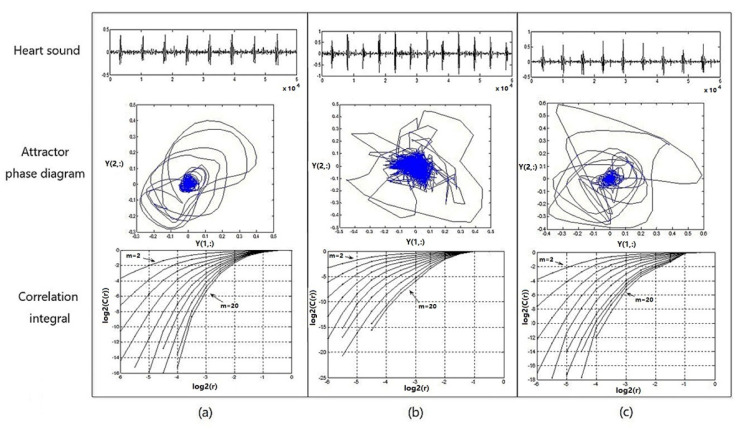
Heart sound waveform, attractor phase diagram, and associated integral distribution diagram for No. 1 test subjects under 3 states: (**a**) resting state—Step 1:A; (**b**) state of motion—Step 2:A; (**c**) recovery state after exercise—Step 3:A.

Figure 9 shows the heart-sound waveform, the attractor phase diagram, and the associated integral distribution diagram of tester numbered 1 in three states. Figure 9a provides a graph representing the heart-sound signals collected in a resting state (Step 1:A); Figure 9b provides a graph representing the heart-sound signals collected in Step 2:A in a state of motion; and Figure 9c provides a graph showing the heart-sound signals collected in Step 3:A after exercise.

It can be seen from Figure 9 that (1) the attractor phase diagrams of heart sounds in the three states are obviously different from each other, indicating that the attractor phase diagram presents significant differences in the distribution area and complexity of the track. The distribution area of the phase–space attractor trajectory in Figure 9b is significantly larger than that in Figure 9a,c, while the order of the phase–space attractor trajectory in Figure 9b is the worst. (2) In the correlation integral distribution diagram, the embedding dimension m gradually increases from 2 to 20, as does the distribution curve when Δm=2. With an increase in the embedding dimension m, the correlation dimension also increases, but with an increase in m, the correlation dimension gradually shows a trend of convergence. The distribution area of the correlation integral distribution curve in Figure 9c is significantly smaller than the curves in Figure 9a,b, and the correlation dimension values are both larger, which indicates that the cardiovascular system has the ability to automatically adjust and presents a stronger chaotic state when the load is increased, thereby providing a positive response to the state of motion.

Therefore, in the process of transitioning from a resting state to a post-exercise recovery state, the chaotic complexity of heart-sound signals, overall, presents a certain trend of decreasing first and then increasing afterwards. This trend indicates that motion has a significant effect on the chaotic characteristics of heart sounds. Moreover, the state of recovery after exercise provides a wealth of information about cardiac regulation.

## 4. Conclusions

In the present paper, using graphic representations, we studied the effects of motion on the characteristics of heart sounds. The main conclusions are as follows:

(1) According to our experiment, when the heart-sound signal changed from a resting state to a state of motion, the sound-direction vector remained basically unchanged, and the similarity distance between the two was very small (=0.0038), which indicates that the heart, as a sound source, had no effect on the acoustic physical characteristics of the sound source itself;
(2) The change of the heart-sound signal from a resting state to a state of motion had little effect on the state-change-trend chart and the difference value of the state change. In the signal of the P channel, the similarity distance between Rest P and Motion P was 0.0011; in the signal of the M channel, the similarity distance between Rest M and Motion M was 0.0006; in the signal of the T channel, the similarity distance between Rest T and Motion T was 0.0038; in the signal of the A channel, the similarity distance between Rest A and Motion A was 0.0013. This result indicates that the difference between similar distances was 0.0032;
(3) The change from a rest to a state of motion can be observed through the amplitude of heart sound, the diastolic heart sound, the systolic period, the frequency characteristics, and the fluctuation of blood pressure. Heart sound amplitude and blood pressure are directly proportional to the heart load, while the diastolic/systolic period of heart sound decreased with an increase in exercise intensity;
(4) Because the size of a heart is clearly greater than the distance from the auscultation point to the heart, the heart as a whole cannot be equivalent to the point source. Thus, only the corresponding local cardiac auscultation area is considered equivalent to the point source, making the four corresponding cardiac auscultation areas equivalent to four different point sources. Consequently, some of the characteristics of heart-sound signals present obvious differences. In the later stage, we will carry out relevant research on heart sound during motion from the perspective of physiology and clinical medicine.

## Figures and Tables

**Table 1:** Effects of motion on the duration of diastolic and systolic heart sounds.

Heart rate (beats/min)	70–80	80–90	90–100	100–110	110–120	120+
Mean systolic period (s)	0.1421	0.1335	0.1171	0.1012	0.0838	0.0571
relative rate of systole	-	6.03%	12.27%	13.58%	17.16%	31.89%
Mean diastolic period (s)	0.3909	0.3502	0.2991	0.2544	0.2371	0.2008
Relative rate of diastole	-	10.41%	14.60%	14.93%	6.79%	15.34%

## Data Availability

The data that support the findings of this study are available upon request from the corresponding author. The data are not publicly available due the fact of privacy restrictions.
